# The British Journal of Dermatology

**Published:** 1888-12

**Authors:** 


*The British Journal of Dermatology.* Edited by MALCOLM MORRIS and H. G. BROOKE. No. 1. Vol. I. November, 1888. London: H. K. LEWIS.

This somewhat shabby-looking publication cannot be said to be premature. British Dermatology—a high-sounding term—ought long ago to have had a periodical of its own, and, in default of anything better, we are glad to welcome this monthly issue of six-and-thirty pages. The communications in this number are from Dr. Thomas Barlow, Mr. Jonathan Hutchinson, and Dr. Unna. If the editors are able to secure for future numbers the co-operation of workers such as these, the journal should be a success.

The reviews of books are well done. This is a department that should be carried on with entire fearlessness, as its work is perhaps the most important branch of journalism, involving as it does the recommendation of books which may have a life-long influence, prejudicial or favourable, on those who so often are induced to obtain them through journal-notices.

A useful "Précis" of current skin-work completes the number. We hope that in future the sheets will be stitched through the folds.

